# Draft genome sequences of *Salmonella enterica* subsp. *enterica* serovar Give sequence type 516 isolated from blood and brain abscess of a child from North India

**DOI:** 10.1128/mra.00841-24

**Published:** 2025-04-11

**Authors:** Paulami Dutta, Subhasree Roy, Gourab Halder, Manisha Ghosh, Arindam Ganai, Archana Angrup, Shanta Dutta

**Affiliations:** 1 Department of Bacteriology, ICMR-National Institute for Research in Bacterial Infections (NIRBI), Kolkata, India; 2 Department of Bioinformatics, ICMR-National Institute for Research in Bacterial Infections (NIRBI), Kolkata, India; 3 Department of Medical Microbiology, Postgraduate Institute of Medical Education and Research (PGIMER), Chandigarh, India; University of Maryland School of Medicine, Baltimore, Maryland, USA

**Keywords:** *Salmonella* Give, invasive non-typhoidal *Salmonella*, draft genome sequencing, comparative genomic analysis, clinical samples

## Abstract

Invasive non-typhoidal *Salmonellae* remains a substantial cause of morbidity and mortality. Here, we present the draft genome sequences of *Salmonella* Give ST516, isolated from blood and brain abscess of a child. The genome sizes are 4,690,901 bp and 4,703,054 bp, assembled in 64 and 67 contigs respectively, and possess *aac(6′)-Iaa*.

## ANNOUNCEMENT

Invasive non-typhoidal salmonellosis can be fatal, particularly in children and elderly adults (1). *Salmonella enterica* subsp. *enterica* serovar Give was previously reported from ruminants and pigs in Quebec (2, 3). Human infection through consumption of contaminated meat products was reported in the US and Europe (4). Here, we report the draft genome sequences of *Salmonella* Give ST516, isolated from a child.

A 3-year-old female child with acute encephalopathy syndrome was admitted to the paediatric ward of PGIMER, Chandigarh, India, in December 2022. The patient’s paired peripheral blood samples were collected before starting antibiotic treatment. A brain abscess specimen was taken from the left-sided subdural abscess in operation theater under general anesthesia. The specimens were collected with parental consent and as a part of the treatment protocol. Samples were inoculated in sheep blood agar (BD BBL) and MacConkey agar (BD, Difco) followed by 16 hours of incubation at 37°C. The isolates were identified as *Salmonella* in MALDI-TOF (Biomerieux) and were sent to ICMR-NIRBI, where they were confirmed as *S*. Give by serotyping (5, 6). The isolates were susceptible to the tested antibiotics as detected by the Kirby-Bauer disk-diffusion method (Table 1). For the isolation of gDNA, bacterial colonies were inoculated into tryptic soy broth (BD, Bacto) and incubated at 37°C for 16 hours. gDNA was extracted using DNeasy blood and tissue kit (Qiagen), quantified in Qubit 3.0 fluorometer. DNA libraries were prepared for single-end sequencing (400 bp chemistry) using Ion Xpress Plus Fragment Library Kit as per the user manual (Thermo Fisher Scientific, TFS). First, the gDNA was sheared using Ion Shear Plus 10× Reaction Buffer and Enzyme Mix II (TFS) followed by adapter ligation and nick repairing. The DNA was size selected using E-Gel Size Select II Agarose Gel as per the NGS library size selection reference (400 bp) and sequenced on the IonTorrent-S5 platform using Ion530 chip (TFS). Read filtering and trimming were performed by BaseCaller module, inbuilt into Torrent-Suite Software (version 5.16.1) (7). Reads were assembled in contigs using the *de novo* assembler SPAdes (version 13.3.0) (8). Contigs were annotated by Prokaryotic Genome Annotation Pipeline (version 6.6) of NCBI (9). The annotated assemblies were investigated for the presence of antimicrobial resistance genes (ARGs), plasmids, and sequence types using ResFinder (4.6.0) (10), PlasmidFinder (2.1) (11), and MLST (2.0) (11), respectively, available at Centre for Genomic Epidemiology (11). The virulence genes were determined using Virulence Factor Database (4.0) (12). Default parameters were used for all the analyses.

**TABLE 1:** Result of antimicrobial susceptibility testing (AST) and summary of genome characteristics of *Salmonella* Give (*n* = 2) clinical isolates

AST and genome characteristic	Result for sample ID:	
	OSS-712	OSS-713
Source of isolation	Blood	Brain abscess
AST profile^*a*^	Amp (S), C (S), T (S), Q (S), Nal (S), Cip (S), 3Cs (S), St (S), G (S), Ak (S), Az (S), AmC (S)	Amp (s), C (s), T (s), Q (S), Nal (S), Cip (S), 3Cs (S), St (S), G (S), Ak (S), Az (S), AmC (S)
Genome size (bp)	4,690,901	4,703,054
No. of reads	983,448	1,118,588
Mean read length (bp)	268	261
No. of contigs	64	67
G+C content (%)	52.22	52.21
*N*_75_	94,979	79,869
*N*_50_	283,930	283,928
Genes (total)	4,589	4,592
CDSs (total)	4,479	4,485
Genes (coding)	4,298	4,311
CDSs (with protein)	4,298	4,311
rRNA (5S, 16S, 23S)	8, 11, 10	8, 9, 10
tRNA	72	71
ncRNAs	9	9
Virulence genes	*fimA*, *pefB*, *safB*, *mgtB*, *misL*, *invA*, *ssaC*, *cdtB*, *pltA*	*fimA*, *pefB*, *safB*, *mgtB*, *misL*, *invA*, *ssaC*, *cdtB*, *pltA*
AMR gene	*aac(6′)Iaa*	*aac(6′)Iaa*
ST type (MLST)	516 (*aroC_84*, *dnaN_11*, *hemD_16*, *hisD_42*, *purE_40*, *sucA_71*, *thrA_4*)	516 (*aroC_84*, *dnaN_11*, *henD_16*, *hisD_42*, *purE_40*, *sucA_71*, *thrA_4*)
GenBank accession number	JAYGAW000000000	JAYGAX000000000
SRA accession number	SRR28409737	SRR28409736

^*a*^ Amp, ampicillin: 10 µg; C, chloramphenicol: 30 µg; T, tetracycline: 30 µg; Q, sulfamethoxazole/trimethoprim: 1.25/23.75 µg; Nal, nalidixic acid: 30 µg; Cip, ciprofloxacin: 5 µg; 3Cs, cefotaxime, ceftazidime, and ceftriaxone: 30 µg each; St, streptomycin: 10 µg; G, gentamicin: 10 µg; Ak, amikacin: 30 µg; Az, azithromycin: 15 µg; AmC, amoxicillin-clavulanic acid: 20/10 µg; and S, susceptible.

**Fig 1:**
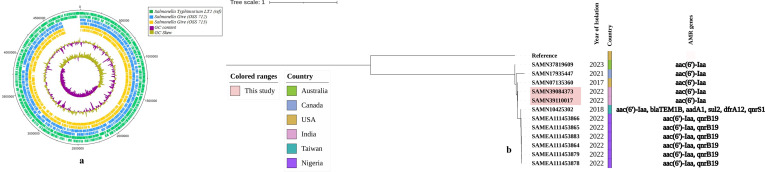
(a) Circular genome of *S*. Give ST516 study strains (OSS 712 and OSS 713), compared with *S*. Typhimurium LT2 (reference strain) plotted from the Artemis tool (version 1.8.0.0) using DNA plotter, with default parameter (14). The outer scale is marked in megabases. The tracks from the outside represent (1) *Salmonella* Typhimurium LT2 forward CDS; (2) *Salmonella* Typhimurium LT2 reverse CDS; (3) *Salmonella* Give (OSS-712) forward CDS; (4) *Salmonella* Give (OSS-712) reverse CDS; (5) *Salmonella* Give (OSS-713) forward CDS; (6) *Salmonella* Give (OSS-713) reverse CDS; (7) %GC plot; and (8) GC skew. (b) WGS-based SNP phylogenetic tree of *S*. Give ST516 study strains (OSS-712 and OSS-713) were compared with *S*. Typhimurium LT2 (reference strain) and global blood isolates (*n* = 10) available at EnteroBase (version 1.2.0). The SNP analysis was done using CSI phylogeny (version 1.4) tool, and the phylogenetic tree was visualized in iTOL (version 6.0).

The genome sizes of OSS-712 and OSS-713 were 4,690,901 bp and 4,703,054 bp, respectively, with an average G+C content of 52%. The genomic features and circular genome plot of the isolates are presented in Table 1; Fig. 1a, respectively. The isolates were pan-susceptible with no notable ARGs, except *aac(6′)-Iaa*, which is a chromosomal, cryptic gene present in most *Salmonella* serovars (13).

The phylogenetic relationship of the study strains was compared by WGS-based SNP analysis with only the available blood isolates (*n* = 10) of *S*. Give ST516 available at EnteroBase (version 1.2.0) (15) in CSI phylogeny (version 1.4) (16). The phylogenetic tree (Fig. 1b) revealed that the Indian isolates were closely related to the USA isolate (SAMN07135360), which was also pan-susceptible. These sequences will serve as references from this region for comparative global genome analysis.

## Data Availability

This whole-genome shotgun project has been deposited in DDBJ/ENA/GenBank under the BioProject PRJNA1056124, and the accession numbers of the sequences are JAYGAW000000000 (OSS-712) and JAYGAX000000000 (OSS-713). The accession numbers of the raw sequences, deposited in the Sequence Read Archive (SRA), are listed in Table 1. The versions described in this paper are the first versions, JAYGAW000000000.1 (OSS-712) and JAYGAX000000000.1 (OSS-713).
